# Trends in the prevalence and care-seeking behaviour for acute respiratory infections among Ugandan infants

**DOI:** 10.1186/s41256-019-0100-8

**Published:** 2019-03-29

**Authors:** Sanni Yaya, Ghose Bishwajit

**Affiliations:** Faculty of Social Sciences, School of International Development and Global Studies, University of Ottawa, 120, University Private, Ottawa, ON K1N 6N5 Canada

**Keywords:** Acute respiratory infections, Care-seeking, Demographic and health survey, Global Health, Infant, Uganda

## Abstract

**Background:**

Acute Respiratory Infections (ARIs) as a group of diseases/symptoms constitute a leading cause of pediatric morbidity and mortality in sub-Saharan Africa where over 10 % of all children die before reaching their fifth birthday. Although the burden of ARIs is highest in the African countries, there is little evidence in the current literature regarding their prevalence and treatment seeking. The objective of this study was therefore to assess the secular trend in the prevalence of ARIs as well as their treatment seeking-behaviour among Ugandan infants.

**Methods:**

This cross-sectional study was based on data from Uganda Demographic and Health Surveys (conducted between 1995 and 2016) on 26,974 singleton infants aged 0–5 months. Mothers (aged 15–49 years) were interviewed to collect information on the prevalence of recent occurrences of fever, cough and dyspnea. The adjusted trend in the prevalence and predictors of ARIs and care seeking were measured by multivariate regression methods.

**Results:**

In 2016, the prevalence of fever, cough and dyspnea was respectively 36.23, 42.55 and 19.27%. The prevalence of all three symptoms has been declining steadily since 1995, and the percentage of children receiving treatment for fever/cough has also more than doubled during the same time. In multivariable analysis, several sociodemographic factors emerged as significant predictors of ARIs including child’s age and high birth order, mother’s age, educational level, occupation, intendedness status of the child, BMI, household wealth status, and place of residency.

**Conclusions:**

The overall prevalence common ARIs (fever, cough, dyspnea) has been declining at a slow but steady rate, however, remains noticeably high in comparison with countries with similar level of per capita GDP in Africa. Findings of this study has important implications for health policy making regarding the prevention of ARIs among infants in the country.

## Introduction

Acute respiratory infections (ARIs) are the leading causes of death among under-5 children especially in Africa, the region that accounts for over two-fifth of all ARI-induced mortalities in the world [1]. The burden of ARIs has declined remarkably in the high-income countries where the ARIs were the most important contributor to infant mortality in the last century [2]. The overall prevalence has declined over the course of the past 4–5 decades, however, ARIs still represent the most prevalent type of infectious diseases even in many developed countries including USA [3, 4]. Led by the strong programmatic efforts to improve child health related indicators within the frameworks of the Millennium Development Goals (MDGs), a great number of countries in Africa have made appreciable strides in terms of preventing the common causes of maternal and child death. Unfortunately, the progress has been uneven across countries with some of the countries experiencing little improvement in child health situation during last two decades e.g. Uganda. An analysis of Uganda Demographic and Health Surveys suggest that there has been a net increase in the prevalence of under-5 mortality in the country between 1995 and 2000 (147.3 deaths per 1000 live births in 1995 Vs 151.5 deaths in 2000) [5]. The findings were hard to account for as data on cause-specific infant mortality rates are not available for Uganda. Given the absence of a functional infant mortality database and surveillance system, information on secular trends in ARIs can aid in interpreting the high under-5 mortality rates in the country.

In Uganda, like in other countries at similar stages of development, high rates of infectious diseases are usually attributed to underdeveloped healthcare systems, growing epidemic of malaria and HIV, seasonal outbreaks of waterborne diseases, socioeconomic inequality in the provision of care, inadequate access to water and sanitation facilities, and environmental pollution [6–11]. The overall situation of public health and healthcare is further exacerbated by political instability, armed conflict, sexual and gender-based crimes that significantly hinder the development efforts and take huge tolls on population health particularly of the vulnerable groups e.g. women and children [12]. Uganda is also a major foreign aid recipient with official development assistance (ODA) generally contributing to around 10% of national budget [13]. While the amount of aid to the healthcare sector has also increased substantially and helped tackle the overall burden of diseases [14], there is no concrete evidence regarding the effectiveness of aid on the known contributors to child mortality such as ARIs and malnutrition (which exert aggravating effect on each other).

Of note, infant mortality rates during the early MDG period have not been sensitive to socioeconomic progress in Uganda which makes the prevention of infectious diseases among children even more challenging [15]. Added to the socioeconomic factors are the concerns surrounding the suboptimal vaccination coverage [16] and poor health-seeking behaviour for children [17–19]. WHO recommends that children manifesting the signs of ARIs e.g. cough accompanied by short, rapid breathing, should be brought to medical attention on urgent basis. Despite the well-documented public health significance of ARIs, a great majority of the countries in sub-Saharan Africa lack country-representative evidence on the prevalence and treatment seeking that are necessary to set up priorities for actions [20]. UpToDate data on key child health related indicators such as ARIs is critical to developing preventive measures and effective intervention tools to achieve childhood mortality related goals. To this end, we conducted this study with the aim of measuring the trends of three common symptoms of ARIs e.g. fever, cough and dyspnea and their care-seeking bahaviour in Uganda during last two decades (1995–2016). We have additionally assessed the sociodemographic patterns in the prevalence of ARIs which can assist in community level targeted interventions.

## Methods

### Setting

The Republic of Uganda is a landlocked nation East Africa sharing frontier with Kenya in the east, Tanzania in the south, Rwanda in the southwest, the Democratic Republic of Congo in the west, and Sudan in the north. Uganda became independent of British colonial rule in October 1962. The country has a population of 41.49 million (as of 2016) living in an area of 241,039 km2. Uganda is divided into 80 administrative districts, which are subdivided into counties, subcounties, and parishes. The economy is based mainly on agricultural activities with coffee being the most important exporting product in terms of revenues. The country is generally food self-sufficient and experienced a flourishing economy following independence. However, the country experiences long-standing political violence and civil unrest with significant bearing on economic, social and healthcare infrastructure.

### Survey and sampling

UDHS are conducted by Uganda Bureau of Statistics (UBOS) in collaboration with the Ministry of Health (MOH) with technical and financial support provided by the Government of Uganda, the United States Agency for International Development (USAID), the United Nations Children’s Fund (UNICEF), and the United Nations Population Fund (UNFPA). The main purpose of these surveys is to provide country-wide data necessary for monitoring and evaluation of population, health, and nutrition programmes and assist in evidence-based health policy making. The surveys are conducted by face-to-face interviews on eligible men (15–54 years) and women (15–49 years) using structured questionnaires containing several components: individual men, women, children (0–59 months), couples and households. Year of surveys and scope of sampling areas were listed in Table 1. The data are made freely accessible in the public domain to all stakeholders. Data for this study were based on women’s questionnaire. More detailed version of the sampling techniques regarding the surveys were published in the final reports [21–24].

### Description of variables

The outcome variables were the recent occurrence of ARI symptoms for the youngest child which were measured by asking the mothers whether or not the child had signs of. .. recently: 1) fever, 2) cough and 3) dyspnea (short, rapid breaths). The answers were categorised as: *Yes* and *No (No/Don’t Know).* Those who responded *Yes* were asked whether or not the child received any treatment. Answer to the questions on treatment were categorised as: *Yes* and *No (No/Don’t Know)*.

Depending on the availability on the datasets, as well their theoretical relationship, the following child and maternal level variables were selected as the potential predictors of occurrences of ARI: Age of child (< 2 months, 2–5 months); Sex of child (Male/Female); Birth order (1st child, 2nd child, 3rd child, 4th or); Mother’s age (15–24, 25–34, > 34); Education (No education, Primary, Secondary/higher); Occupation (Service/skilled manual, Agriculture/self-employed, Not working/other); Religious affiliation (Catholic, Islam/others); BMI* (Underweight, Normal weight, Overweight, Obese); Household wealth status** (Poor, Non-poor); Child was wanted (No, Yes); Cooking fuel** (Unclean, Clean); Residency (Rural, Urban).

*Defined as Underweight = < 18.5 kg/m^2^, Normal weight = 18.5–24.9 kg/m^2^, Overweight = 25–29.9 kg/m^2^, Obese= > 30 kg/m^2^ [25]. **Defined as Clean = Electricity, Biogas, Liquefied petroleum gas; Unclean = Kerosene, wood. ***Defined in terms of the wealth quintiles calculated based on household possession of durable goods (e.g. TV, Refrigerator) The scores are then categorised into quintiles, with higher quintiles representing better wealth status. For this study wealth quintiles were merged into two categories: Q1 + Q2 = Poor, Q3 + Q4 + Q5 = Non-poor [26].

### Data analysis

Data were analysed with SPSS 24. Datasets were cleaned and merged in order to perform pooled analysis. Women who are not married and whose last childbirth were not singleton were excluded from the analysis. Normality tests are conducted and absence of correlation was verified by using variance inflation factor method (VIF). Following that, the dataset was accounted for the cluster sampling design, sampling strata and weight by using complex survey mode. Sample characteristics were described by percentages with 95% CIs. Trends in the prevalence of fever, cough and dyspnea and of children receiving treatment for fever and cough were presented as bar charts (information on treatment seeking for dyspnea was not available). Odds ratios of recent occurrence of fever, cough and dyspnea (short, rapid breath) were measured using binary logistic regression techniques. Lastly, odds ratios of treatment seeking for fever/cough across the survey years were measured using binary logistic regression analysis. All tests were two-tailed and was considered significant at alpha value of 5%.

### Ethical clearance

Ethical approval was not necessary for this study as the data were secondary and are available in public domain in anonymised form.

## Results

### Sample characteristics

Basic sociodemographic characteristics of the sample population were summarised in Table 2. In brief, majority of the children were below two months of age and male with birth order of four or higher. Regarding maternal and household characteristics, a greater proportion were within the age bracket of 25–34 years, had primary level education, employed in agriculture, followers of Christianity, of normal body weight. Majority of the mothers were from non-poor households (3rd-5th wealth quintile), reported last child as unintended, used unclean fuel for cooking, and were rural residents.

### Trend in the prevalence of and treatment-seeking behaviour for fever, cough and dyspnea among Ugandan infants

**Fig. 1:**
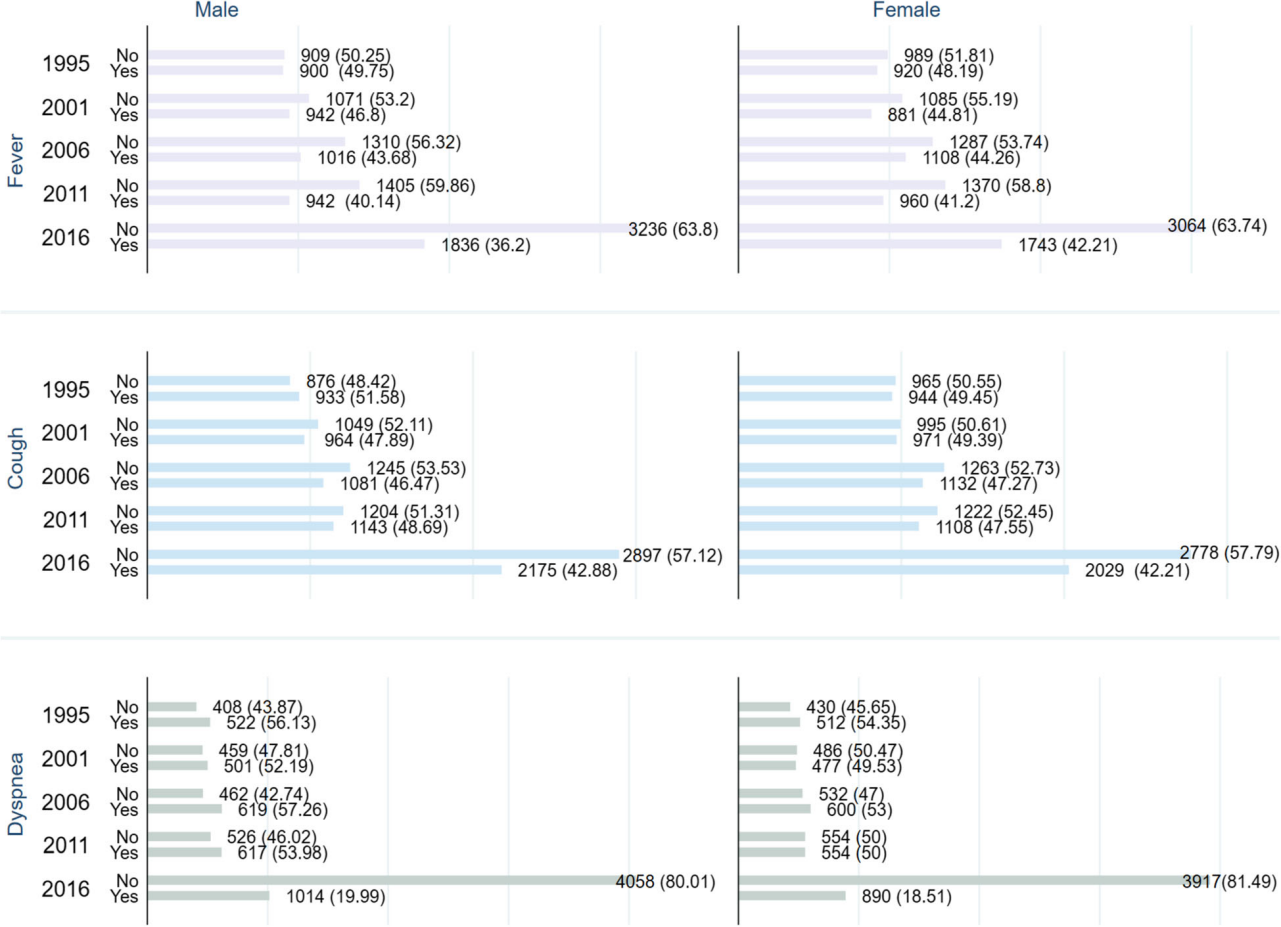
Secular trend in the prevalence of fever, cough and dyspnea among Ugandan infants between 1995 and 2016

**Fig. 2:**
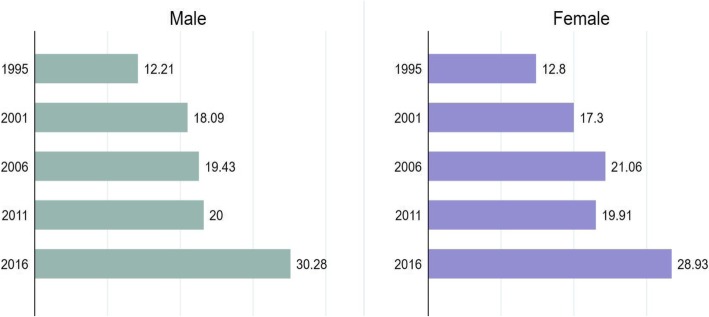
Percentage of infants receiving treatment for fever/cough (*n* = 14,334)

In 2016, the prevalence of fever, cough and dyspnea was respectively 36.23, 42.55 and 19.27% in contrast to the pooled prevalence of 41.7, 46.27 and 34.77% in the aforementioned order. Figure 1 shows that the proportion of children suffering from fever, cough and dyspnea have declined considerably among both boys and girls since 1995. The progress has been most noticeable for the prevalence of dyspnea with about three-fold decline in 2016 in contrast with 1995. The percentage of children receiving treatment for fever/cough has also more than doubled during the same time (Fig. 2).

**Table 1:** List of surveys used

Year	No. of clusters	Coverage	Field work	Response rate (%)
1995	303	All of the districts but Kitgum and Pader	March–August.	95.8
2000–01	298	All of the districts but Amuru, Bundibugyo, Gulu, Kasese, Kitgum, and Pader.	September 2000–March 2001	93.9
2006	368	All the districts	May–October	94.7
2011	404	All the districts	June–December	93.8
2016	697	All the districts	June–December	97

**Table 2:** Sample characteristics (*n* = 26,974)

Variables	Category	n	Percentage	Lower 95% CI	Upper 95% CI
Year					
	1995	3722	13.8	12.6	14.4
	2001	3992	14.8	14.5	15.7
	2006	4720	17.5	16.4	18.6
	2011	4667	17.3	16.3	18.5
	2016	9872	36.6	34.2	37.3
Child-level variables					
Age of child					
	< 2 months	22,415	83.1	82.4	83.7
	2–5 months	4559	16.9	16.3	17.6
Sex of child					
	Male	13,568	50.3	49.6	51.0
	Female	13,406	49.7	49.0	50.4
Birth order	1st child	4936	18.3	17.6	18.9
	2nd child	4559	16.9	16.3	17.5
	3rd child	4019	14.9	14.4	15.5
	4th or higher	13,460	49.9	49.0	50.8
Maternal and household -level variables					
Mother’s age					
	15–24	9765	36.2	35.4	36.9
	25–34	11,734	43.5	42.7	44.3
	> 34	5476	20.3	19.7	20.9
Education					
	No education	5071	18.8	17.8	19.9
	Primary	16,319	60.5	59.3	61.6
	Secondary/higher	5584	20.7	19.6	21.9
Occupation					
	Not working	6069	22.5	21.3	23.8
	Service/skilled manual	6258	23.2	22.1	24.3
	Agriculture/self employed	14,620	54.2	52.5	56.0
Religious affiliation					
	Catholic	22,658	84.0	82.7	85.2
	Islam/others	4316	16.0	14.8	17.3
BMI					
	Underweight	2239	8.3	7.7	9.0
	Normal weight	20,608	76.4	75.4	77.3
	Overweight	3156	11.7	10.9	12.4
	Obese	998	3.7	3.3	4.1
Household wealth status					
	Poor	10,547	39.1	37.4	40.9
	Non-poor	16,427	60.9	59.1	62.6
Child was intended					
	No	16,186	56.3	55.3	57.3
	Yes	11,787	43.7	42.7	44.7
Cooking fuel					
	Unclean	26,893	99.7	99.5	99.8
	Clean	81	0.3	0.2	0.5
Residency					
	Urban	7040	26.1	24.6	27.7
	Rural	19,799	73.4	71.8	74.9

### Multivariate analysis measuring the trend of ARIs and treatment-seeking

**Fig. 3:**
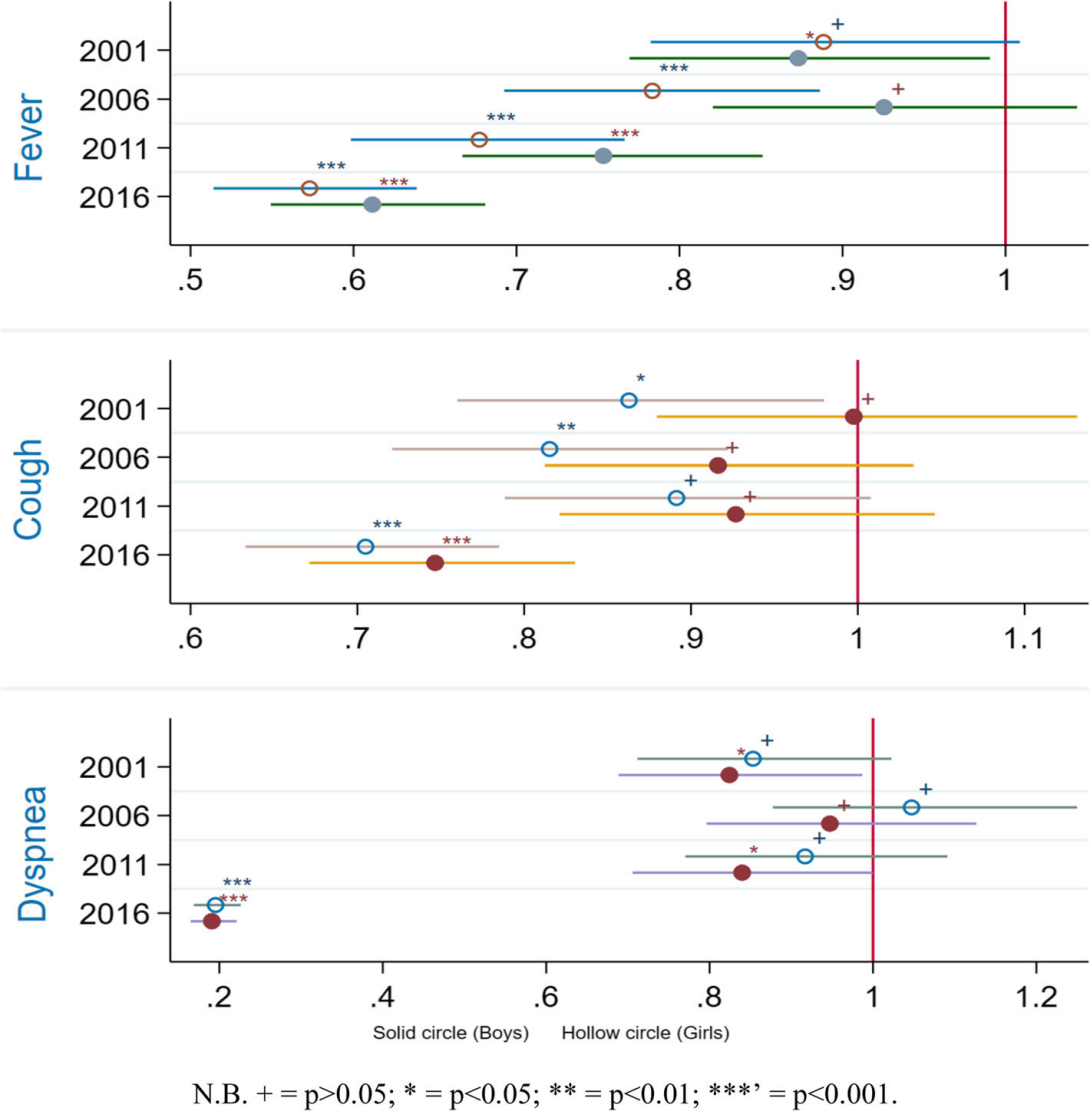
Trends in the odds ratios of ARIs among Ugandan infants 1995–2015

**Fig. 4:**
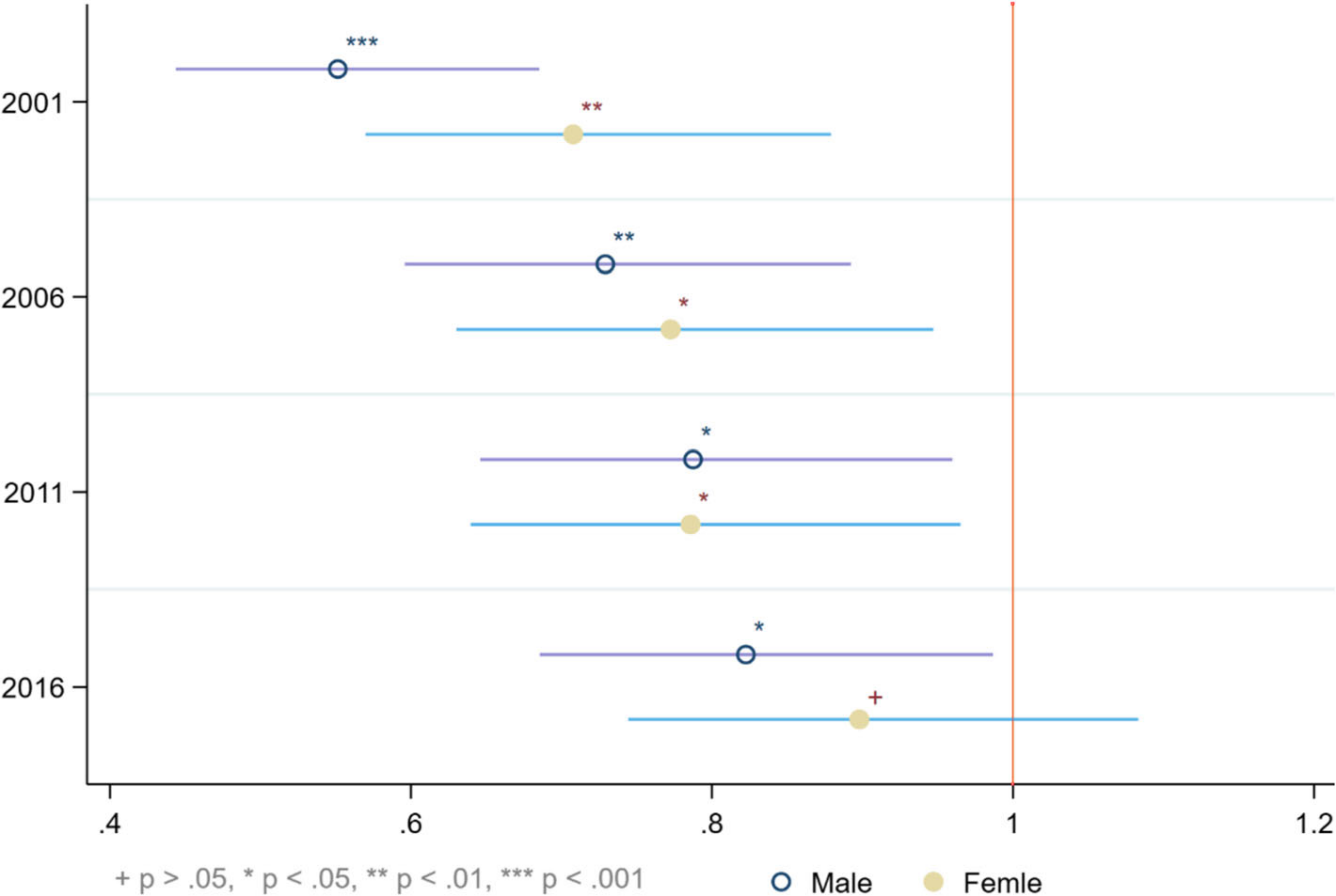
Trends in odds ratios of not receiving treatment for fever/cough. 1995–2015

Multivariate analysis was performed to assess the trend of decline and in ARIs (Fig. 2) and their treatment-seeking behaviour (Fig. 3). As of 2016, the odds of fever among boys and girls were respectively 0.58 and 0.62 times, that of cough respectively 0.71 and 0.76 times and dyspnea about 0.2 times lower in comparison with their 1995 levels. Regarding treatment-seeking for fever/cough, the odds of not receiving any treatment were significantly lower for all the survey years except for girls in 2016 (Fig. 4).

**Table 3:** Odds ratios of recent occurrence of fever, cough and dyspnea among Ugandan infants

	Fever			Cough			Dyspnea		
	Overall**	Boys	Girls	Overall**	Boys	Girls	Overall**	Boys	Girls
Child-level variables									
Age of child (< 2 months)									
2–5 months	1.305*	1.252*	1.366*	1.282*	1.323*	1.241*	1.366*	1.305*	1.436*
Birth order (4th)									
1st child	0.711*	0.803*	0.632*	1.053	1.068	1.055	0.754*	0.812	0.700*
2nd child	0.720*	0.770*	0.673*	0.972	1.018	0.924	0.765*	0.900	0.641*
3rd higher	1.057	1.135	0.987	0.942	0.996	0.887	0.994	1.001	0.986
Maternal and household -level variables									
Age of mother (15–24)									
25–34	1.410*	1.287*	1.530*	1.087	0.950	1.237*	1.402*	1.253	1.551*
> 34	1.184*	1.155	1.204*	1.038	0.977	1.096	1.195*	1.138	1.244*
Educational level (No education)									
Primary	1.212*	1.164	1.261*	1.136	0.980	1.317*	1.834*	1.647*	2.057*
Secondary/higher	1.233*	1.224*	1.240*	1.074	0.911	1.266	1.399*	1.194	1.662*
Occupation (Not working)									
Service/skilled manual	0.965	0.975	0.952	0.859*	0.859*	0.854*	1.020	0.976	1.067
Agriculture/other	0.969	0.972	0.964	1.040	1.020	1.042	0.828*	0.770*	0.884
Religion (Catholic)									
Islam/others	0.826*	0.734*	0.928	0.997	0.976	1.017	1.086	1.140	1.042
BMI (Obese)									
Underweight	1.933*	2.061*	1.827*	1.436*	1.291	1.593*	0.842	0.983	1.057
Normal weight	1.458*	1.623*	1.314*	1.079	1.012	1.152	1.139	1.239	1.050
Overweight	1.029	1.084	0.985	0.940	0.825	1.084	1.100	1.161	0.822
Child was intended (No)									
Yes	0.780*	0.719*	0.845*	0.842*	0.846*	0.835	0.885	0.958	1.051
Wealth status (Non-poor)									
Poor	1.119*	1.106	1.131*	0.978	0.967	0.993	0.987	0.932	0.998
Residency (Rural)									
Urban	0.684*	0.627*	0.743*	0.905*	0.841*	0.971	0.933	0.873*	1.091

N.B. * = significant at *p* < 0.05. ** = adjusted for sex

### Predictors of fever, cough, and dyspnea among Ugandan infants

As shown in Table 3, several sociodemographic factors appeared to be significantly associated with recent occurrences fever, cough and dyspnea among Ugandans infants. For instance, those in the age group of 2–5 months had respectively 1.30, 1.28 and 1.36 times higher odds of suffering the fever, cough and dyspnea. Compared with first-borns, second- and third-borns had relative lower odds of suffering from fever and dyspnea. Children of mothers from higher age brackets and with higher education also had higher odds of suffering from fever and dyspnea. Mothers employment in service/skilled job was associated with lower odds cough among both boys (OR = 0.85) and girls (0.85). Mothers who were under- and normal weight had children with higher odds of suffering from fever and cough. Children who were intended were less likely to suffer from fever and cough compared with those who are reported as unintended. Children from poor households had 1.11 times higher odds of suffering from fever, and those from urban areas had respectively 0.68 and 0.90 times lower of suffering from fever and cough.

## Discussion

Our findings suggest that during last two decades the prevalence of fever, cough and dyspnea have declined considerably among Ugandan infants with the prevalence of fever being higher among girls than boys and that of cough and dyspnea being slightly higher among boys. Despite this appreciable progress, the prevalence rates remain remarkably higher in comparison with the most recent findings from Nigeria (3.8% as of 2013) [27, 28], Ethiopia (7% as of 2011) [29], Rwanda (4% as of 2010) [30]. Apart from reducing the prevalence of these ARIs, measurable progress has been achieved in increasing the use of professional treatment such as visiting healthcare centres as well. Overall, the percentage of infants for whom treatment was sought for fever/cough has more than doubled since 1995. However, the prevalence was marginally higher among girls than boys: 12.21% in 1995 Vs 30.28% in 2016 among boys in contrast with 12.8% in 1995 Vs 28.93% in 2016 among girls. These findings indicate a general improvement of child health situation in the context of ARIs which is likely, in part, to be attributable to the MDG-led efforts.

Uganda’s performance in achieving the MDGs has been described as ‘Impressive’ [31] on achieving 6 of 14 measured targets (for which adequate data were available) and missing another 3 by narrow margins [32], including that of reducing under-5 mortality. While lack of disease specific infant mortality data makes it hard to measure to what extent the high prevalence of ARIs might be responsible behind this suboptimal performance, our findings indicate that there is clearly a lot remains to be accomplished, especially in reducing the prevalence and achieving universal care for ARIs.

Another important finding that emerged from the analysis is the sociodemographic pattern in the prevalence in the distribution of ARIs. The prevalence did not appear to differ noticeably across sex, whereas age difference was a significant predictor of all three and birth order of fever and dyspnea. Higher age group (2–5 months) was found to be a risk factor while lower birth order as a protective factor. Higher birth order is a known risk factor of child malnutrition and higher mortality rates in low-income countries especially among women with high fertility rates [33, 34]. In low-income settings, each additional child can result in higher competition for resources such as nutrition and healthcare and consequently leading to poorer health status. Findings of our study adds to the current literature the evidence that higher birth order is associated with higher odds of ARI symptoms as well.

Apart from child level factors, several maternal and household level characteristics were found to be significant predicting the occurrence of ARIs including mother’s age, education, occupation, nutritional status, intendedness of the child, household wealth, residency. Previous studies have pointed out the role of maternal demographic and socioeconomic factors on child’s health outcomes. Of the factors that significantly predicted the ARIs the one that is particularly remarkable is the intendedness status of the child. Although evidence on adverse health consequences of unwanted childbirths on women’s health have been well-documented in the literature [35, 36], that on child health outcome is relative scarce. Given the high fertility and low contraception rates among Ugandan women [37–39], it is suggestible that child health promotion programs pay particular attention on the addressing the risk factors of unwanted pregnancy. Last but not least, urban residency was found to be a protective factor against fever and cough especially among boys. Urban-rural disparity in health and healthcare is widespread across Africa that needs to be dealt with in order to achieve comprehensive health gains especially in terms of reducing child morbidity and mortality rates.

Child malnutrition and infectious diseases represent two major risk factors for child mortality in African countries. Taking into consideration the fact that inadequate access to improved water and sanitation (WASH) remains a major public health issue in Africa, healthcare systems need to regard ARIs as an urgent imperative owing to the high prevalence and widespread distribution of the risk factors. Insofar as child health related targets are concerned in the post-MDG era, provision of quality evidence on ARIs and their associated factors are of paramount importance for designing effective prevention and intervention strategies. From this perspective, insights generated by our study can play crucial role in current policy making and implementation especially in the context of countries like Uganda characterised by high child poverty, malnutrition and mortality rates. Future researches should focus on investigating the broader sociocultural and macroeconomic factors that underlie the poor healthcare seeking behaviour for ARIs among Ugandan children.

As far as we are concerned, this is the first study to report progress on the prevalence of ARI symptoms and their treatment-seeking in a sub-Saharan African country. We used data from large, county-representative surveys that are considered as reliable sources of information on key health indicators in developing countries. Data were analysed using rigorous statistical methods and interpreted in light of the status quo in order to facilitate communication for future researches and policy actions. Besides the important contribution, we have several limitations to declare that needs to be taken into consideration while interpreting the findings. Firstly, the outcome variables were measured based on mothers’ responses and hence there is no guarantee that the responses were based on medical diagnosis. As such, it is possible that some children were suffering from diseases which had the manifestation similar to ARIs. Therefore, the results should be construed with caution as the symptoms of ARIs may not indicative of the diseases. As most of the variables were self-reported, the findings remain subject to recall and reporting bias [40, 41], as some individuals are more likely to give socially acceptable answers to certain questions. We were also unable to include several variables that are strong predictors of child health. The data were cross-sectional and hence no causal relationship can be inferred from the associations [42, 43].

## Conclusion

In conclusion, there has been a significant decline in the prevalence of ARI symptoms among Ugandan infants. This finding is in line with the achievement of reduced under-five mortality during the MDG period. Nonetheless, it should be noted that the prevalence of ARIs has been falling at a slow rate and still remain remarkably higher compared with most other countries in sub-Saharan Africa. As the findings further indicate, improvement is needed in promoting the treatment seeking behaviour as well. Significant sociodemographic disparities were observed in the distribution of ARIs which should be addressed in order to achieve a more even progress in the post-MDG era. Continued international funding and collaboration in combination with new momentum from the Sustainable Development Goals can greatly benefit Uganda’s development trajectory especially in its fight against poverty and child mortality in the coming years.

## Abbreviations

ARIs: Acute respiratory infections
DHS: Demographic and Health Survey
LMICs: Low-income countries
